# Molecular Markers in Upper Urothelial Carcinoma Associated to Balkan Endemic Nephropathy. Aristolochic Acid as the Major Risk Factor of the Worldwide Disease

**DOI:** 10.1100/tsw.2009.162

**Published:** 2009-12-16

**Authors:** Ljubinka Jankovic Velickovic, Takanori Hattori, Vladisav Stefanovic

**Affiliations:** ^1^ Institute of Pathology, Faculty of Medicine, University of Nis, Serbia; ^2^ Department of Pathology, Shiga University of Medical Science, Ohtsu, Japan; ^3^ Institute of Nephrology, Faculty of Medicine, University of Nis, Serbia

**Keywords:** Balkan endemic nephropathy, aristolochic acid nephropathy, p53, Ki-67 index, upper urothelial cancer, molecular markers

## Abstract

The role of aristolochic acid in the etiology of Balkan endemic nephropathy (BEN) and associated upper urothelial carcinoma (UUC) was recently confirmed. The aim of this study was to determine the marker(s) specific for BEN-associated UUC. A total of 82 patients with UUC (38 from the BEN region and 44 control tumors) were included in the study. The Ki-67 index in BEN tumors correlated with the grade and multifocality (*p* < 0.05), but in regression analysis, only the grade of BEN tumor. The p53 index was significantly higher in BEN than in control tumors (*p* < 0.05), as well as the alteration of p53 (*p* < 0.05). BEN low-stage tumors, tumors without limphovascular invasion (LVI), and tumors of the renal pelvis had a higher p53 index than the control tumors (*p* < 0.05, 0.01, 0.05, respectively). The Ki-67 index was higher in control tumors with high-stage and solid growth than in BEN UUC (p < 0.050, 0.005). The Ki-67 correlated with the grade, growth, stage, LVI, and multifocality of UUC on the best way, but not with the group. In regression analysis, only multifocality of UUC had predictive influence on Ki-67 activity (*p* < 0.001). P53 correlated with the grade, growth, and group (*p* < 0.05). This investigation identifies the p53 pathway as the specific cell cycle marker involved in BEN-associated UUC.

### INTRODUCTION

Balkan endemic nephropathy (BEN) is a chronic tubulointerstitial disease prevalent in Bosnia and Herzegovina, Bulgaria, Croatia, Romania, and Serbia. In addition to renal disease, an increased number of upper urothelial carcinomas (UUC) has been observed in the foci of BEN. Carcinoma may occur alone or in combination with BEN[1,2]. Urothelial malignancies of the renal pelvis and ureter are significantly more frequent, up to 100 times, in endemic than in nonendemic areas. They tend to cluster in families affected with BEN, indicating an association between these diseases and, probably, a common etiologic agent[2]. Tumors develop usually later than the interstitial nephropathy. Their incidence increases with age at the time of diagnosis and with a longer survival, and is higher in females.

The hypothesis that aristolochic acid (AA), a nephrotoxic and carcinogenic plant alkaloid derived from *Aristolochia* species, could be a potential etiologic factor for BEN was formulated as early as 1969, based on epidemiologic, environmental, and experimental research[3]. It was observed that *A. clematitis*, a plant native to the endemic region, often grows in cultivated fields where its seeds, containing AA, commingle with wheat grain during the annual harvest. As bread, the dietary staple of the region, is prepared traditionally from flour made from locally grown wheat, residents of the endemic region that consume home-baked bread may be exposed to toxic amounts of AA over time.

AA is the cause of Chinese herb nephropathy (now called aristolochic acid nephropathy, or AAN), a rapidly progressive interstitial fibrosing renal disease with frequent urothelial malignancies[4]. The similarity of several clinical and histological features between AAN and BEN has led to the hypothesis that AA could be the cause of BEN[5]. The etiologic role of aristolochia in BEN was proposed some 40 years ago by Ivic[3]. Recently, Grollman et al. demonstrated that (1) DNA adducts derived from AA are present in renal tissues of patients with documented BEN, (2) these adducts can be detected in transitional cell cancers (TCC), and (3) A:T $\rightarrow$ T:A transversions dominate the p53 mutational spectrum in the upper urinary tract malignancies found in this population[6]. It is concluded that dietary exposure to AA is a significant risk factor for BEN and its attendant TCC[6,7].

Biomarkers are biomolecules that serve as indicators of biological and pathological processes, or responses to a drug treatment. Due to the complexity of structure and function of protein biomarkers and lack of high sensitivity, specificity, and reproducibility, the combination of different biomarkers, such as performed in this study, could validate a combination of markers for diagnosis and prognosis of urothelial cancer[8,9].

There are no studies on biological markers in UUC associated with BEN/AAN. The aims of this study were to evaluate the relationship between phenotypic characteristics and molecular markers, p53, p16, cyclin D1, HER-2, and proliferative Ki-67 activity in specimens of UUC in the BEN region; to correlate expression of these molecular markers in UUC in the BEN region and control population; and to determine the marker(s) that is the specific indicator of BEN. Due to the demonstrated AA etiology of BEN, a worldwide disease, markers of AA-induced UUC were determined.

### PATIENTS AND METHODS

#### Patient Population

We studied 82 consecutive patients with UUC who had undergone nephroureterectomy with removal of the bladder cuff. Extended lymphadenectomy was not routinely performed. All cases of UUC were diagnosed at the Institute of Pathology, Faculty of Medicine, University of Nis. The analysis was done on 65 pelvic and 17 ureteral tumors. Patients were divided into two groups: 38 patients were from endemic settlements, villages along the South Morava River basin (BEN tumors), and 44 control subjects included rural and city populations free of BEN. In seven tumors collected from the BEN region, tubulointerstitial lesions similar to BEN were observed.

The histological sections were processed from tissue fixed in 10% formalin by standard techniques, and stained with haematoxylin and eosin (H&E). H&E-stained slides were used to assess histological grade (low and high grade)[10], pathologic stage (pT)[11], growth of tumor (papillary/solid), and lymphovascular invasion (LVI). The authors compared low-stage nonmuscle invasive tumors (pTa-pT1) and high-stage muscle invasive (pT2-pT4) tumors[12].

### Immunohistochemistry and Scoring

Tumors were analyzed using the mouse monoclonal antibody against p53 (Pab 1801, IgG1/ Newcastle), p16 (Clone 6H12, IgG2b/ Newcastle), cyclin D1 (P2D11F11, IgG2a/ Newcastle), HER-2 (Code A 0485/ Dako), and Ki-67 [(MIB-1 (8), isotope IgG1, kappa/ Dako)] at dilution 1:50, 1:40, ready to use, 1:300, 1:100, respectively, and a standard avidin-biotin immunoperoxidase complexes detection system, according to the manufacturer's protocol (Dako LSAB2R system-HRP).

Before quantifying the immunohistochemical results, the technique quality was assessed and those areas with greater positivity were selected, avoiding peripheral area measurement, necrosis, or artifact.

Slides were reviewed independently by three investigators. Interobserver discrepancies were resolved using a double-headed microscope. Only nuclear expression was recorded for p53, p16, cyclin D1, and Ki-67. The number of distinctly positive tumor cell nuclei was counted under high power (×400) using a 10×10 eyepiece grid. In total, 1000 tumor cells were assessed. The number of positive nuclei was expressed as a percentage of all tumor cell nuclei counted. In this way, we defined p53, cyclin D1, and Ki-67 indices. Regarding p53, tumors showing immunoreactivity of more than 10% of the tumor cell nuclei were considered altered[13].

The staining protocol for p16 nuclear protein includes heterogeneous, homogeneous, and negative findings. A tumor was considered to have a normal heterogeneous p16 pattern if it had relatively weak nuclear staining with considerable differences in nuclear intensity, including many negative cells. Strong p16 staining was considered if the majority of the malignant cells had intensive p16 nuclear expression and p16 negative tumor cells were rare. A tumor was termed p16 negative if no malignant cells had positive staining. A tumor with or without overexpression of p16 was categorized as altered[14].

For testing HER-2 (C-erbB2) status, we used the HercepTest scoring system devised by DAKO. HER-2 cell membrane–specific immunoreactivity was scored by estimating the percentage of positive tumor cells as follows: score 0, no immunoreactive cells; score +1, positivity in <5% cancer cells; score +2, positivity in 5–50% cancer cells; and score +3, positivity in >50% of cancer cells. The specimens were considered HER-2 positive when the score was ≥2+.

### Statistical Analysis

For purposes of analysis, the pathological tumor stage (low vs. high), grade (low vs. high), growth pattern (papillary vs. solid), LVI (yes vs. no), and clinical parameters (sex [M vs. F], localization [pelvis vs. ureter], multifocality [yes vs. no]) were evaluated as dichotomized variables. The Fisher's exact test and the $\chi^2$ test were used to evaluate the expression of p16 and HER-2 with pathological parameters (stage, grade, growth, lymphovascular invasion) and multifocality of tumors. Other molecular markers (p53, cyclin D1, Ki-67) are expressed as means ± standard deviation, and statistical significance between these groups was estimated according to the Student's t-test for unpaired samples. Correlation between molecular markers (p53, p16, cyclin D1, HER-2, Ki-67) and conventional pathological parameters was analyzed by Pearson correlation and regression analysis. The result was considered statistically significant if $p < 0.05$.

All analyses were performed with the SPSS statistical package (SPSS version 10.0 for Windows).

## RESULTS

### Clinical Features in UUC

There were 47 males (57%) and 35 females (43%) in the study, with relation M:F = 1.3:1. The median age of the 82 UUC patients was 63.3 ± 10.6 years (range: 22–87). Tumor localization was more frequent on the left side (51 [62%] vs. 31 [38%]). Among the 82 patients with UUC, 67 (82%) had solitary tumors, while 15 (18%) patients had multifocality of tumors at the time of presentation.

### Pathological Features in BEN Tumors

The number of patients with low- and high-grade UUC in the BEN region was 13 (34%) and 25 (66%), respectively. The pathological stage was low in 15 (39%) and high in 23 (61%) tumors. Tumors had papillary growth in 10 (26%) and solid in 28 (74%) specimens. LVI was detected in 11 (29%) and multifocality in six (16%) BEN tumors. Morphological analysis of BEN tumors showed significant association between pathological stage and grade ($\chi^2 = 7.32$, $p < 0.01$), pathological stage and growth pattern ($\chi^2 = 9.33$, $p < 0.005$), as well as pathological stage and LVI ($\chi^2 = 5.98$, $p < 0.05$) (Table 1).

**TABLE 1**
**Morphological Finding and Pathological Stage in BEN Tumors**

| | Low stage N (%) | High stage N (%) | *p* < |
|---|---|---|---|
| Grade | | | |
| Low grade | 9 (24) | 4 (10) | |
| High grade | 6 (16) | 19 (50) | 0.01 |
| Growth | | | |
| Papillary | 8 (21) | 2 (5) | |
| Solid | 7 (19) | 21 (55) | 0.005 |
| LVI | | | |
| Yes | 1 (3) | 10 (26) | |
| No | 14 (37) | 13 (34) | 0.05 |
| Multifocality | | | |
| Yes | 2 (5) | 4 (11) | |
| No | 13 (34) | 19 (50) | N.S. |

N.S., no significance.

### Evaluation of Immunohistochemical Staining in BEN Tumors

Normal urothelium of the kidney pelvis and ureter showed heterogeneous expression of p16 (wild type) and absence of p53 (wild type).

The mean p53 labeling index was 15.8 ± 21.1 (SD) (range: 0–84.4) and 23 specimens (61%) were positive for p53 expression (Figs.1a,b). Immunohistochemical analysis of p16 showed that 18 (47%) tumors had altered p16 expression; i.e., 14 (37%) UUC were p16 negative, while four (10%) tumors had strong homogeneous nuclear staining (Fig. 1c). A normal heterogeneous p16 staining pattern was detected in 20 (53%) UUC. The mean labeling index for cyclin D1 was 8.9 ± 9.1 (SD) (range: 0–27.4) and 24 specimens (63%) were positive for cyclin D1 expression (Fig. 1d). Investigation of the HER-2 status of 38 BEN UUC showed that 18 (47%) tumors were HER-2 positive (≥2+ staining), while only seven (18%) were classified as 3+ positive (Fig. 1e). Twenty (53%) BEN tumors were HER-2 negative (score ≤1). The mean Ki-67 labeling index was 9.7 ± 9.2 (SD) (range: 0.1–41.7) (Fig. 1f).

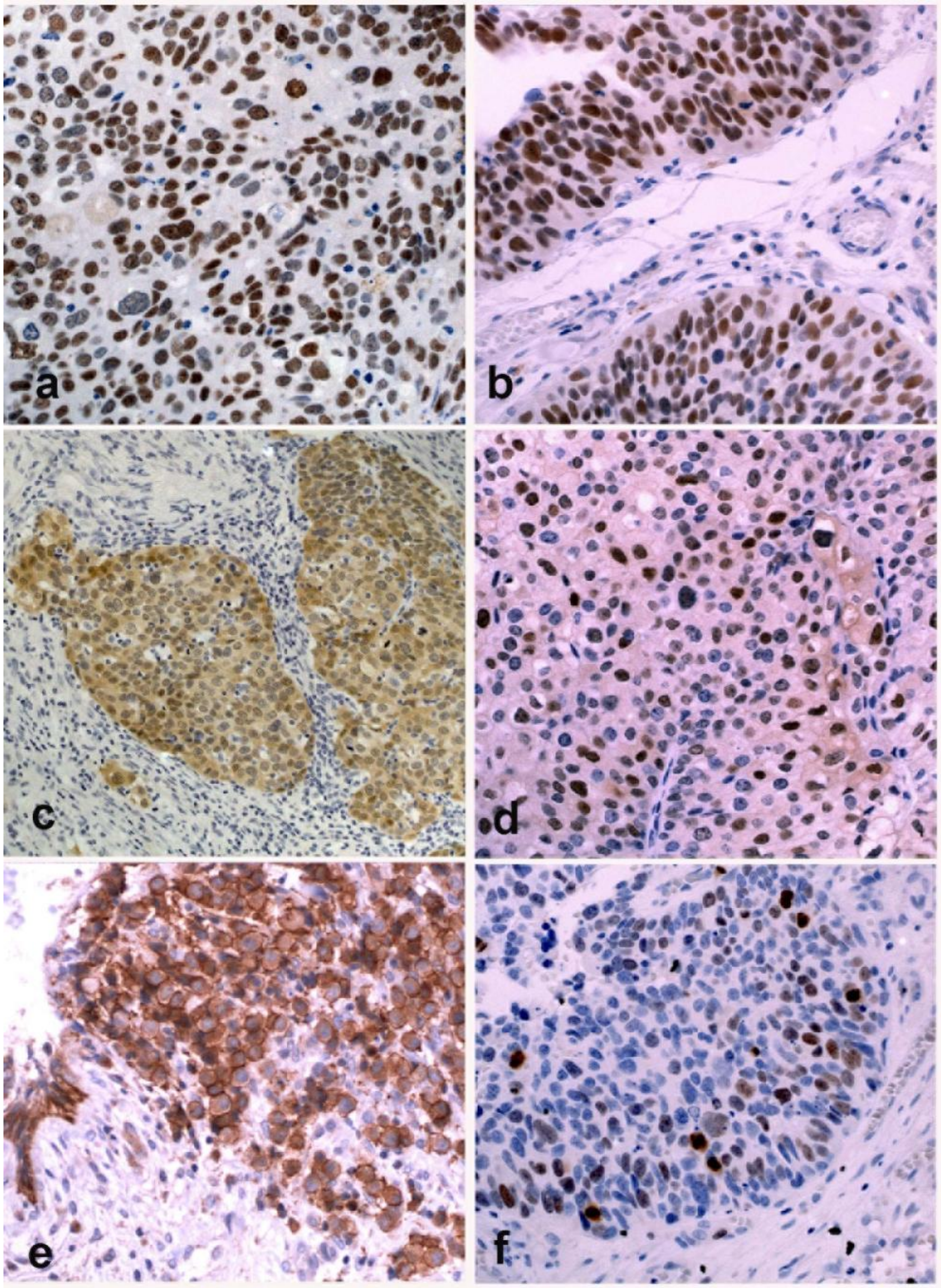

**FIGURE 1.** The representative immunohistochemical staining of molecular markers in BEN-associated urothelial carcinoma, with strong nuclear p53 activity in high-grade (a) and low-stage tumors (b); homogeneous p16 nuclear and cytoplasm expression (c), nuclear cyclin D1 staining in high-grade tumor (d). HER-2–positive tumor cells classified as 3+ positive (e). Proliferative Ki-67 expression in a high-grade tumor (f) (original magnification: ×400)

Expression of p53, p16, cyclin D1, and HER-2 was not significantly associated with the conventional pathological parameters and multifocality in BEN tumors. Investigation of the proliferative Ki-67 labeling index showed that Ki-67 significantly correlated with the grade and multifocality ($p < 0.05$) of BEN tumors (Table 2). Regression analysis of the pathological features and Ki-67 in BEN tumors indicated a statistically significant difference ($F = 3.408$, $p < 0.05$), where grade had predictive influence on Ki-67 activity ($\beta = 0.508$, $t = 2.994$, $p < 0.005$).

**TABLE 2**
**Ki-67 Index in Relation to Grade, Stage, Growth, LVI, and Multifocality of BEN Tumor**

| Pearson Correlation | | Grade | Stage | Growth | LVI | Multifocality |
|---|---|---|---|---|---|---|
| Ki-67% | **r** | 0.345 | –0.115 | –0.106 | 0.124 | 0.295 |
| | ***p*** < | 0.05 | N.S. | N.S. | 0.229 | 0.05 |

### Comparison of Cell Cycle–Related Markers in BEN and Control UUC

Tables 3–6 show relationships between conventional pathological parameters and immunohistochemical staining of p53, p16, cyclin D1, HER-2, and Ki-67 in BEN and control UUC.

The p53 labeling index was significantly higher in BEN than in control tumors (15.8 ± 21.1 [SD] vs. 6.88 ± 15.66 [SD], $p < 0.05$). p53 was altered (more than 10% of the tumor cell nuclei) in 16/38 (42%) BEN tumors (Fig. 1a) and 9/44 (20%) control tumors with significant difference ($\chi^2 = 4.51$, $p < 0.05$). Also, BEN tumors with low-stage (Fig. 1b), with no LVI, and localization in the renal pelvis had a significantly higher p53 index than control tumors ($p < 0.05$, 0.01, 0.05, respectively) (Table 3).

There was no difference in the expression of p16, HER-2, and cyclin D1 between BEN and control UUC, as well as in their relation to the conventional pathological parameters and localization – renal pelvis or ureter (Tables 4 and 5). The Ki-67 labeling index was significantly higher in control tumors with the high-stage ($p < 0.05$) and solid growth ($p < 0.005$) than in BEN tumors with the same morphological characteristics; and localization did not have an influence on proliferative Ki-67 activity (Table 6). There was no significant difference in the Ki-67 index between BEN and control UUC (15.8 ± 21.1 [SD] vs. 6.88 ± 15.66 [SD]).

A possible correlation between p53, p16, cyclin D1, HER-2, Ki-67, and standard pathological features and group was investigated. Pearson's coefficient of correlation showed that tumor suppressor p53 correlated with the grade ($p < 0.05$), growth ($p < 0.05$), and with the group ($p < 0.05$). Tumor suppressor p53 was identified as a specific indicator of the UUC arising in patients with BEN. Proliferative marker Ki-67 correlated with the grade ($p < 0.005$), growth ($p < 0.01$), stage ($p < 0.05$), LVI ($p < 0.05$), and multifocality ($p < 0.001$) of UUC on the best way, but not with the group (Table 7). However, in the regression model, only multifocality of UUC had a predictive influence on Ki-67 expression ($\beta = 0.338$, t = 3.335, $p < 0.001$).

### DISCUSSION

The p53 gene product (located at 17p12), called "the guardian of the genome", plays a role in cell cycle regulation at the G1/S-phase checkpoint, DNA repair, and apoptosis[15]. Normally, p53 levels in a cell are low because the protein is rapidly degraded by its inhibitor, $MDM_2$ protein. p53 may be knocked out by deletion, by mutation, or by the action of inhibitor $MDM_2$ (which binds p53 and targets it for degradation).

On the other hand, cyclin D1 is an important positive regulator of the G1/S transition and is expressed in the early G1 in response to mitogenic signals[16]. Forced overexpression of G1 phase cyclins can overcome control of the G1/S checkpoint, leading to uncontrolled proliferation.

The amplification of HER-2 or overexpression of its product in urothelial carcinomas has been associated with tumor grade, tumor stage, and the patient's outcome[17]. The level of expression and the prognostic significance of HER-2 protein in urothelial cancer vary among different studies, with some revealing no prognostic relevance and other suggesting a better or worse prognosis[8,9].

**TABLE 3**
**Pathological Variables of UUC and p53 Index in Control and BEN Tumors**

| UUC – p53% | N | Mean | SD | SE | *p* < |
|---|---|---|---|---|---|
| Low grade | | | | | |
| Control | 27 | 4.5 | 5.72 | 1.1 | |
| BEN | 13 | 9.87 | 14.99 | 4.15 | N.S. |
| High grade | | | | | |
| Control | 17 | 10.66 | 24.11 | 5.84 | |
| BEN | 25 | 18.86 | 23.37 | 4.67 | N.S. |
| Low stage | | | | | |
| Control | 15 | 3.47 | 4.68 | 1.21 | |
| BEN | 15 | 14.52 | 16.21 | 4.18 | 0.05 |
| High stage | | | | | |
| Control | 29 | 8.64 | 18.87 | 3.5 | |
| BEN | 23 | 16.61 | 24.09 | 5.02 | N.S. |
| Papillary growth | | | | | |
| Control | 24 | 3.35 | 5.75 | 1.17 | |
| BEN | 10 | 11.74 | 14.73 | 4.66 | N.S. |
| Solid growth | | | | | |
| Control | 20 | 11.11 | 21.92 | 4.9 | |
| BEN | 28 | 17.23 | 23.02 | 4.35 | N.S. |
| LVI Yes | | | | | |
| Control | 12 | 14.02 | 28.13 | 8.12 | |
| BEN | 11 | 15.06 | 20.99 | 6.33 | N.S. |
| LVI No | | | | | |
| Control | 32 | 4.2 | 5.68 | 1 | |
| BEN | 27 | 16.08 | 21.55 | 4.1 | 0.01 |
| Multifocality yes | | | | | |
| Control | 9 | 2.97 | 4.61 | 1.53 | |
| BEN | 6 | 10.09 | 16.64 | 6.79 | N.S. |
| Multifocality No | | | | | |
| Control | 35 | 7.88 | 17.32 | 2.92 | |
| BEN | 32 | 16.85 | 21.9 | 3.87 | N.S. |
| Pelvis | | | | | |
| Control | 37 | 5.39 | 9.86 | 1.62 | |
| BEN | 28 | 18.75 | 22.32 | 4.22 | 0.05 |
| Ureter | | | | | |
| Control | 7 | 14.78 | 33.0 | 12.47 | |
| BEN | 10 | 7.51 | 15.31 | 4.84 | N.S. |

N.S., no significance.

This study demonstrated that analysis of p53 provides additional information about UUC from the BEN region. BEN tumors have a significantly higher p53 index than control tumors. Correlation analysis of expression-investigated molecular markers p53, p16, cyclin D1, HER-2, Ki-67, and group showed that only tumor suppressor p53 was a specific indicator of group. This investigation detected the p53 pathway as the specific cell cycle marker involved in BEN-related UUC.

**TABLE 4**
**Pathological Variables of UUC and Expression of p16 and HER-2 in Control and BEN Tumors**

| Variables/Groups | N | p16 | | *p* < | HER-2 Score | | *p* < |
|---|---|---|---|---|---|---|---|
| | | Normal | Altered | | ≤1 | ≥2 | |
| Low grade | | | | | | | |
| Control | 27 | 14 | 13 | | 10 | 17 | |
| BEN | 13 | 7 | 6 | N.S. | 8 | 5 | N.S. |
| High grade | | | | | | | |
| Control | 17 | 5 | 12 | | 9 | 8 | |
| BEN | 25 | 13 | 12 | N.S. | 12 | 13 | N.S. |
| Low stage | | | | | | | |
| Control | 15 | 6 | 9 | | 5 | 10 | |
| BEN | 15 | 9 | 6 | N.S. | 6 | 9 | N.S. |
| High stage | | | | | | | |
| Control | 29 | 13 | 16 | | 14 | 15 | |
| BEN | 23 | 11 | 12 | N.S. | 14 | 9 | N.S. |
| Papillary growth | | | | | | | |
| Control | 24 | 9 | 15 | | 10 | 14 | |
| BEN | 10 | 6 | 4 | N.S. | 5 | 5 | N.S. |
| Solid growth | | | | | | | |
| Control | 20 | 10 | 10 | | 9 | 11 | |
| BEN | 28 | 14 | 14 | N.S. | 15 | 13 | N.S. |
| LVI Yes | | | | | | | |
| Control | 12 | 4 | 8 | | 8 | 4 | |
| BEN | 11 | 6 | 5 | N.S. | 5 | 6 | N.S. |
| LVI No | | | | | | | |
| Control | 32 | 15 | 17 | | 11 | 21 | |
| BEN | 27 | 14 | 13 | N.S. | 15 | 12 | N.S. |
| Multifocality yes | | | | | | | |
| Control | 9 | 4 | 5 | | 3 | 6 | |
| BEN | 6 | 2 | 4 | N.S. | 2 | 4 | N.S. |
| Multifocality No | | | | | | | |
| Control | 35 | 15 | 20 | | 16 | 19 | |
| BEN | 32 | 18 | 14 | N.S. | 18 | 14 | N.S. |
| Pelvis | | | | | | | |
| Control | 37 | 15 | 22 | | 16 | 21 | |
| BEN | 28 | 15 | 13 | N.S. | 16 | 12 | N.S. |
| Ureter | | | | | | | |
| Control | 7 | 4 | 3 | | 3 | 4 | |
| BEN | 10 | 5 | 5 | N.S. | 4 | 6 | N.S. |

N.S., no significance; $\chi^2$ test was performed.

**TABLE 5**
**Pathological Variables of UUC and Cyclin D1 Index in Control and BEN Tumors**

| UUC – Cyclin D1 Index | N | Mean | SD | SE | *p* < |
|---|---|---|---|---|---|
| Low grade | | | | | |
| Control | 27 | 12.54 | 12.19 | 2.34 | |
| BEN | 13 | 6.62 | 7.07 | 1.96 | N.S. |
| High grade | | | | | |
| Control | 17 | 10.91 | 10.96 | 2.65 | |
| BEN | 25 | 10.06 | 9.97 | 1.99 | N.S. |
| Low stage | | | | | |
| Control | 15 | 11.85 | 11.76 | 3.03 | |
| BEN | 15 | 11.05 | 8.82 | 2.27 | N.S. |
| High stage | | | | | |
| Control | 29 | 11.94 | 11.77 | 2.18 | |
| BEN | 23 | 7.47 | 9.25 | 1.93 | N.S. |
| Papillary growth | | | | | |
| Control | 24 | 10.74 | 11.27 | 2.3 | |
| BEN | 10 | 8.04 | 6.33 | 2 | N.S. |
| Solid growth | | | | | |
| Control | 20 | 13.31 | 12.19 | 2.72 | |
| BEN | 28 | 9.19 | 10.03 | 1.89 | N.S. |
| LVI Yes | | | | | |
| Control | 12 | 11.45 | 10.82 | 3.12 | |
| BEN | 11 | 5.71 | 7.97 | 2.4 | N.S. |
| LVI No | | | | | |
| Control | 32 | 12.08 | 12.08 | 2.13 | |
| BEN | 27 | 10.18 | 9.40 | 1.8 | N.S. |
| Multifocality yes | | | | | |
| Control | 9 | 13.94 | 11.52 | 3.84 | |
| BEN | 6 | 7.52 | 8.53 | 3.48 | N.S. |
| Multifocality No | | | | | |
| Control | 35 | 11.38 | 11.76 | 1.98 | |
| BEN | 32 | 9.14 | 9.35 | 1.65 | N.S. |
| Pelvis | | | | | |
| Control | 37 | 11.72 | 11.37 | 1.87 | |
| BEN | 28 | 9.05 | 9.34 | 1.76 | N.S. |
| Ureter | | | | | |
| Control | 7 | 12.94 | 13.89 | 5.25 | |
| BEN | 10 | 8.45 | 9.03 | 2.85 | N.S. |

N.S., no significance; t-test was performed.

Some authors suggested that accumulation of p53 protein in 10% or more of the tumor cell nuclei strongly correlates with mutations in the p53 gene[13]. Our investigation showed that 42% the BEN tumors and 20% of the control tumors had alteration of nuclear p53 immunoreactivity, with statistically significant difference.

**TABLE 6**
**Proliferative Activity Ki-67 in Control and BEN UUC in Relation to Pathological Variables**

| UUC – Ki-67 Index | N | Mean | SD | SE | *p* < |
|---|---|---|---|---|---|
| Low Grade | | | | | |
| Control | 27 | 8.58 | 7.88 | 1.51 | |
| BEN | 13 | 5.33 | 5.11 | 1.41 | N.S. |
| High Grade | | | | | |
| Control | 17 | 13.51 | 10.04 | 2.43 | |
| BEN | 25 | 11.93 | 10.09 | 2.01 | N.S. |
| Low Stage | | | | | |
| Control | 15 | 5.07 | 4.49 | 1.16 | |
| BEN | 15 | 10.96 | 12.29 | 3.17 | N.S. |
| High Stage | | | | | |
| Control | 29 | 13.29 | 9.51 | 1.76 | |
| BEN | 23 | 8.83 | 6.65 | 1.38 | 0.05 |
| Papillary growth | | | | | |
| Control | 24 | 5.49 | 5.39 | 1.1 | |
| BEN | 10 | 11.28 | 12.53 | 3.96 | N.S. |
| Solid growth | | | | | |
| Control | 20 | 16.48 | 8.88 | 1.98 | |
| BEN | 28 | 9.10 | 7.90 | 1.49 | 0.005 |
| LVI Yes | | | | | |
| Control | 12 | 15.54 | 8.75 | 2.52 | |
| BEN | 11 | 11.44 | 7.14 | 2.15 | N.S. |
| LVI No | | | | | |
| Control | 32 | 8.59 | 8.46 | 1.49 | |
| BEN | 27 | 8.95 | 9.95 | 1.91 | N.S. |
| Multifocality Yes | | | | | |
| Control | 9 | 17.07 | 11.06 | 3.68 | |
| BEN | 6 | 15.87 | 13.66 | 5.57 | N.S. |
| Multifocality No | | | | | |
| Control | 35 | 8.81 | 7.71 | 1.3 | |
| BEN | 32 | 8.51 | 7.88 | 1.39 | N.S. |
| Pelvis | | | | | |
| Control | 37 | 10.91 | 9.68 | 1.59 | |
| BEN | 28 | 9.99 | 9.05 | 1.71 | N.S. |
| Ureter | | | | | |
| Control | 7 | 8.30 | 3.39 | 1.28 | |
| BEN | 10 | 8.81 | 10.08 | 3.19 | N.S. |

N.S., no significance; t-test was performed.

Krasteva et al.[18] screened 90 Bulgarian BEN patients for p53 gene mutations and found that tumor suppressor gene mutations were present in BEN patients. The results obtained are in support of their hypothesis that p53 gene alterations are possibly involved in BEN genetic pathways. Also, Grollman et al. demonstrated AA adducts associated with p53 mutation in UUC from a BEN region in Croatia[6].

**TABLE 7**
**Pearson Correlation Analysis of Molecular Markers with Conventional Morphological Parameters and Group**

| Molecular Markers | Significance | Grade | Growth | LVI | Multifocality | Stage | Group |
|---|---|---|---|---|---|---|---|
| p53% | r | 0.249 | 0.234 | 0.117 | –0.131 | 0.082 | 0.236 |
| p53% | *p* < | 0.05 | 0.05 | N.S. | N.S. | N.S. | 0.05 |
| Ki-67% | r | 0.280 | 0.273 | 0.241 | 0.339 | 0.197 | –0.045 |
| Ki-67% | *p* < | 0.005 | 0.01 | 0.05 | 0.001 | 0.050 | N.S. |

N.S., no significance

In AAN, there is an increased incidence of UUC. Rodent models of AAN may be used to study biotransformation of AA and repair of AL-DNA adducts and, importantly, to validate the use of AL-DNA adducts as biomarkers of disease. The murine model will be particularly useful for determining genetic susceptibility to AAN and, by implication, to BEN[19].

As in AAN, patients with BEN are at increased risk for the development of UUC after kidney transplantation. Thus, 33.3% of patients with BEN developed UUC, compared with a 0.67% prevalence of urinary tract tumors among transplanted patients with other causes of end-stage renal disease[20].

Our investigation showed a significant difference in p53 expression between BEN and control tumors that was connected with the stage of UUC and renal pelvis localization. BEN low-stage UUC have a higher p53 index than control superficial tumors. We did not find any difference in p53 activity between high-stage BEN and control tumors. Renal pelvis UUC from endemic settlements had a significantly higher p53 index than control; the opposite was found in tumors of the ureter, however, without statistical significance.

Mutations in the p53 gene were frequently found in invasive UUC as well as in high-grade superficial UUC (including carcinoma *in situ*, the putative precursor of invasive UUC), whereas these mutations were rare in well-differentiated superficial UUC[21,22].

Our comparative study of E-cadherin expression in UUC between patients from BEN and nonendemic areas showed that BEN low-grade and low-stage tumors have important reduction of E-cadherin expression. The growth pattern had a predominant influence on E-cadherin expression in BEN tumors, as previously described[23]. Decreased E-cadherin expression is indicative for invasion and loss of local control. It is of particular interest to predict recurrence in patients with low-grade noninvasive tumors[24]. Deregulation of p53 and E-cadherin in low-stage UUC from BEN areas may be indicative for a potentially more aggressive tumor.

Low incidence of p53 mutations in Ta/T1 bladder tumors may suggest that p53 alterations in superficial cancer are related to a more aggressive phenotype and a higher risk of recurrence. Loss of p53 is a late event in tumor development. The earlier stages of tumor evolution are not prevented by p53[25,26,27].

Different tissues have different susceptibility to p53 deficiency. The urothelium is less susceptible to tumorigenesis during p53 deficiency than fast-renewing cells, such as lymphocytes and fibroblasts[28]. Cycling cells in any given environment are subjected to a different kind of stress, and that could compromise DNA synthesis. Normal human cells elicit the p53-dependent and p53-independent responses, resulting in suppression of cyclin-dependent kinase 2 (CDK2) activities and inhibiting centrosome duplication. Centrosome amplification is one of the causes of carcinoma *in situ*, and is associated with a loss or mutational inactivation of p53. In human cells lacking functional p53, when DNA synthesis is blocked, p53-independent pathways suppress CDK2 kinase activity and do not allow centrosome reduplication[29,30,31].

On the other hand, investigation of proliferate Ki-67 activity showed that control high stage, as well as control tumors with solid growth, had a higher Ki-67 index than BEN tumors with the same morphological characteristics. There was no difference in Ki-67 activity between BEN low-stage and control low-stage tumors, as well as depending on localization (renal pelvis or ureter).

In BEN tumors with extreme cell atypia, a higher degree of apoptosis was shown, but proliferation does not dramatically increase with UUC progression to high grade. Chronic ischemia in surrounding tissue may facilitate apoptosis in BEN-related tumors[32]. Investigation of the pathogenesis of BEN has suggested that apoptosis in tubular epithelia has a part in the silent development of the disease[33,34].

Our earlier analysis of the morphological characteristics of UUC in BEN and nonendemic regions showed that the best characteristic that differentiated them was growth pattern; i.e., solid growth for BEN tumors and papillary for control tumors[35]. In this study, control UUC with solid growth had a higher Ki-67 index than BEN solid tumors. In UUC with a papillary configuration, there was no difference in proliferate Ki-67 activity.

In conclusion, investigation of multiple molecular markers indentifies tumor suppressor p53 as an indicator of the group, where BEN UUC had a higher p53 index than control tumors. Differentiation of BEN tumors had predictive influence on Ki-67 activity, and their proliferation was lower with stage progression, as well as with solid growth in comparison with control tumors.

### ACKNOWLEDGMENTS

This work was supported by a grant, No 145004, from the Ministry of Science and Technological Development of the Republic of Serbia.
